# Impact of extraction method on the chemical composition and antibacterial potential of *Salvia fruticosa* essential oil

**DOI:** 10.1186/s12906-026-05376-5

**Published:** 2026-05-02

**Authors:** Nashwa Hashad, Hanan G. Sary, Mohamed T. Khazaal, Fatma A. Moharram

**Affiliations:** 1 Department of Pharmacognosy, Faculty of Pharmacy, Capital University (Formerly Helwan University), Ein Helwan, Cairo 11795 Egypt; 2 Department of Pharmacognosy, Faculty of Pharmacy, Ain-Shams University, Cairo, Egypt; 3 Department of Pharmaceutical Chemistry, College of Pharmacy, Kuwait University, Kuwait, Kuwait; 4 Department of Botany and Microbiology, Faculty of Science, Capital University (Formerly Helwan University), Ein Helwan, Cairo 11795 Egypt

**Keywords:** Antibacterial, Biofilm, Essential oils, Gas chromatography, *Salvia fruticose*, Time kill

## Abstract

**Background:**

*Salvia fruticosa* (Lamiaceae) is traditionally used to treat gastrointestinal (GIT) issues, stomach and abdominal pain, and ulcers. In this work, we aimed to extract essential oils (EOs) from the aerial parts of *S*. *fruticosa*, a plant commonly found in Egypt, and to evaluate their antibacterial activity against GIT pathogens.

**Methods:**

Fresh samples’ essential oils (EOs) were extracted using hydrodistillation (HD) and headspace (HS) techniques, while dried samples’ EOs were obtained *via* supercritical fluid (SF) extraction. GC/MS analysis was used to analyze the EOs. Their antibacterial activity was tested against *Clostridium perfringens* ATCC 13124, *Listeria monocytogenes* ATCC 7644, *Staphylococcus aureus* ATCC 25923, *Enterococcus faecalis* ATCC 29212, *Escherichia coli* ATCC 8739, and *Salmonella enterica* ATCC 14028 through agar diffusion, microwell dilution, time-kill, and biofilm formation methods.

**Results:**

The SF method yielded the highest EO (0.6 mL/100 g). In HD and SF-EOs, oxygenated compounds were predominant, accounting for 68.74% and 41.71%, respectively, while non-oxygenated compounds were most common in HS-EOs at 53.64%. Eucalyptol was the main component in both HD (43.87%) and HS-EOs (35.67%), whereas *iso-*caryophyllene (17.07%) and 13-epi-manoyl oxide (12.52%) were key in SF-EOs. Both HD and SF EOs showed kinetics similar to the bacterial control, indicating their potential as effective antimicrobials. Significantly, these EOs exhibited strong bactericidal activity against GIT pathogens, including *C*. *perfringens* (MBC: 500 µg/mL), *L*. *monocytogenes* (MBC: 125 µg/mL), *S*. *aureus* (MBC: 31.25 µg/mL), *E*. *faecalis* (MBC: 125 µg/mL), *E*. *coli* (MBC: 500 µg/mL), and *S*. *enterica* (MBC: 250 µg/mL). The reductions in colony-forming units (CFU/mL) were 3.8, 3.94, 7.76, 6.2, 3.7, and 5.47 log10 CFU/mL for each pathogen, with effects lasting 24 h at concentrations of 2× MIC. The MICs ranged from 31.25 to 250 µg/mL for HD and from 3.91 to 250 µg/mL for SF-EOs, demonstrating their superior antibacterial activity against GIT pathogens compared to previous research.

**Conclusion:**

*S*. *fruticose* EO includes both oxygenated and non-oxygenated volatile compounds and exhibits potent antibacterial effects. They are recommended as bioactive agents for the treatment of gastrointestinal infections, although further research is required to verify their safety for clinical use.

**Supplementary Information:**

The online version contains supplementary material available at 10.1186/s12906-026-05376-5.

## Introduction

The human gastrointestinal tract (GIT) harbors diverse microorganisms that are essential for health, aiding digestion, immune regulation, and protection against microbes. Their long coevolution with the host led to a symbiotic relationship that influences immune responses [1]. Disruptions, or dysbiosis, caused by infections, antibiotics, or lifestyle factors can lead to gastrointestinal issues such as diarrhea, inflammatory bowel disease, and irritable bowel syndrome [1, 2], underscoring the importance of a balanced microbiome for immune health. Additionally, the gut is an entry point for food-borne bacterial pathogens; these pathogens need to interact with the microbiota to colonize the gut, disrupting its balance. The host’s immune responses are adjusted to tolerate commensal bacteria while defending against infections [3]. Pathogenic bacteria such as *Escherichia coli*, *Clostridium perfringens*, and *Salmonella enterica* are concerning due to links with acute and chronic illnesses. Microorganisms form biofilms to protect from stress, facilitate communication, increase virulence, and support growth. They have defense, sustainability, and survival mechanisms that resist environmental challenges, immune responses, and antibiotics [4]. Several gastrointestinal pathogens form biofilms, leading to chronic, recurrent infections less responsive to antibiotics. Managing these infections often requires combination therapy, higher doses, or agents that can break down the biofilm matrix. Understanding how biofilms form in GI pathogens is vital for developing treatments. Future therapies may focus on preventing or disrupting biofilms or improving antibiotic penetration. Conventional treatments involve antibiotics, which are effective but can cause side effects such as disrupting the gut microbiome, fostering resistance, and leading to recurrent infections. Thus, there’s increasing interest in alternative therapies. EOs have activity against GIT infections [5–10] due to their biocidal [9, 10] and anti-inflammatory effects [11], making them promising for new treatments without promoting resistance. They are hydrophobic, complex mixtures of volatile compounds, mainly terpenoids, classified into oxygenated and non-oxygenated groups [12, 13]. Their composition depends on factors like extraction, location, environment, plant maturity, part used, and genetics [14, 15]. Many medicinal plants, including Salvia, produce EOs [16]. The genus Salvia (Lamiaceae) comprises around 900 species, including shrubs, perennial herbs, and annuals. These plants are distributed globally, with notable presence in Central and South America, Central Asia, the Mediterranean, tropical Africa, and eastern Asia [17]. Salvia species are widely used in folk medicine due to their therapeutic properties, which include antibacterial, spasmolytic, and hemostatic effects, making them popular in traditional remedies [18]. *Salvia fruticosa* Mill., also known as Greek sage, with synonyms such as *S. triloba* L., *S*. *libanotica* Boiss. and Gaill., is a perennial aromatic shrub native to the Eastern Mediterranean and North Africa. Due to its value in EOs production, it has been introduced globally [19]. Historically, *S. fruticosa* has been used in folk medicine for various ailments [20], with the infusion of its leaves particularly popular among Palestinians for treating gastrointestinal issues, stomach and abdominal pains, ulcers, headaches, rheumatism, colds, coughs, and influenza [21–23]. In several countries, it is also employed for rheumatic pain relief, as a sedative, carminative, and stomachic [6, 19, 24]. Rich in EOs, *S. fruticosa* has attracted scientific interest for the extraction and analysis of its biological activities using various methods. EOs from Egyptian *S*. *fruticosa* were obtained mainly *via* hydrodistillation (HD) [25–28]; although some studies explore supercritical extraction [29, 30] and a single study on headspace extraction [31], HD remains the dominant global method [19]. Its EOs have been studied for antimicrobial [26, 32, 33], antifungal [32, 34], anti-inflammatory [35], antitrypanosomal [25], and anticancer properties [36], as well as for cholinergic cognitive-enhancing effects [33, 37].

We investigated, for the first time, how the extraction method affects the physical and chemical properties of *S*. *fruticosa* EOs, using three different techniques and analyzing them by Gas Chromatography-Mass spectrometry (GC/MS). We also examined their potential to inhibit pathogens linked to GIT infections, exploring their possible use as alternative treatments for bacterial GIT issues.

## Materials and methods

Aerial parts of *S*. *fruticosa* Mill. (*S. triloba* L.), without flowers, were collected from El-Shikh Zoaid, Sinai, Egypt, in September 2024. In accordance with Egypt’s collection laws and local protected area regulations, the plant was collected after obtaining permission from the authorities responsible for protected areas. It was authenticated by Prof. Abduo M. Hamed, a plant ecology expert (Faculty of Science, Al-Azhar University boys, Cairo). A voucher sample (12Sfr1/2025) was stored in the Herbarium of the Pharmacognosy Department (Faculty of Pharmacy, Capital University (Formerly Helwan University), Egypt.

### Essential oils extraction

#### Hydrodistillation (HD)

250 g of the fresh aerials were used to extract EOs as described in the previous work [38]. Detailed method represented in the supplementary data.

#### Supercritical fluid extraction (SF)

160 g of the dried aerial parts l were used to extract EOs according to the previous work [38]. The detailed method is represented in the supplementary data.

The EOs percentages obtained from HD and SF were reported in mL/100 g of aerial parts [38].

#### Dynamic head-space GC/MS analysis

Fresh aerial parts were used to extract EOs according to the previous work [38]and [39]. The detailed method is represented in the supplementary data.

#### Gas chromatography-mass spectrometry (GC-MS) analysis

The analysis was performed using a Shimadzu GC-MS-QP2010 (Tokyo, Japan). An Rtx-1MS column (30 m x 0.25 mm i.d. x 0.25 μm film thickness, Restek, USA) with a split-splitless injector was utilized. The initial column temperature was set at 45 °C for 2 min (isothermal), then increased to 300 °C at a rate of 5 °C/min and maintained at 300 °C for 5 min (isothermal). The flame ionization detector was operated at 280 °C. The flow rate of the carrier gas (Helium) is 1.41 mL/min. The MS ion source was maintained at 200 °C, and the interface at 280 °C, with electron ionization at 70 eV, scanning from 35 to 500 amu. An oil sample (1 µL) was injected in split mode (1:15). EO components were identified by comparing Kovats’ retention indices (RI) with those of *n*-alkane standards (C8-C28) and by matching MS spectra with NIST and Wiley databases (similarity index > 90%).

### Antibacterial activity

#### Material

*Clostridium perfringens* ATCC 13124, *Listeria monocytogenes* ATCC 7644, *Staphylococcus aureus* ATCC 25923, *Enterococcus faecalis* ATCC 29212, *Escherichia coli* ATCC 8739, and *Salmonella enterica* ATCC 14028. They were obtained from the American Type Culture Collection (ATCC, USA). Mueller-Hinton agar (MHA) and broth (MHB), DMSO were obtained from Sigma-Aldrich (USA), while antibiotic discs (6.0 mm, Oxoid, Thermo Fisher Scientific, USA) were used.

#### Susceptibility test

Using the agar well-diffusion method, we performed the test according to guidelines from the Clinical and Laboratory Standards Institute [40] and [41]. We tested four Gram-positive bacteria (*C*. *perfringens* ATCC 13124, *L*. *monocytogenes* ATCC 7644, *S*. *aureus* ATCC 25923, and *E. faecalis* ATCC 29212) and two Gram-negative bacteria (*E*. *coli* ATCC 8739 and *S*. *enterica* ATCC 14028). We use different antibiotics with various mechanisms of action as reference drugs, while 10% DMSO serves as a negative control. We created a 100 µL suspension with 1 × 10^5^ cells of each reference strain (optical density, OD600 = 0.2) and spread it onto MHA plates. After the agar solidified, we used a 0.6 cm diameter drill bit to create wells and added 50 µL of the tested sample at concentrations of 50, 1, and 0.5 mg/mL. We then refrigerated the plates to facilitate diffusion of the tested samples [42] and incubated them at 37 °C for 24 h. For *C*. *perfringens*, we incubated them in an anaerobic jar.

We measured the antimicrobial activity of the extracts by the diameter of the zones of inhibition (ZOI) in millimeters.

#### Determination of minimum inhibitory concentrations (MIC)

For HD and SF, the assay was conducted using a broth microdilution method, following the guidelines of Balouiri et al. [43] and [40]. The detailed method was represented in the supplementary data.

#### Determination of the minimum bactericidal concentration (MBC)

Using the broth dilution method, as outlined in the [40] guidelines, we determined the results. The detailed method was represented in the supplementary data.

#### Time kill test

According to the methods reported by Foerster et al. [44] and Khazaal et al. [45], the detailed methods are provided in the supplementary data.

#### Anti-biofilm formation quantitative assay

The experiment followed the methods outlined by Kang et al. [46] and Salem et al. [47], with minor modifications (see supplementary data).

#### Statistical analysis

Statistical methods included ANOVA followed by Dunnett’s post-hoc test using GraphPad Prism 5 (GraphPad Inc., CA, USA), and Duncan’s post-hoc test with IBM SPSS version 20 software. We used these methods to compare the decrease in bacterial colony-forming units (CFU/mL), optical density (OD) for viability, and biofilm percentage for each sample relative to the untreated control, with number of replicates for each analysis (*n* = 3).

## Results

Deciding on the best method for extracting EOs from fragrant plants is complex due to various factors [48]. The HD is the most common technique; however, it has its downsides, including the polymerization and isomerization of delicate components [49]. On the other hand, solvent-free (SF) methods are eco-friendly and produce high-quality EOs with minimal degradation in a short duration [50]. SF techniques also offer benefits, including high diffusion rates, increased oil yields, and reduced viscosity. Additionally, the head apace solid-phase microextraction (HS-SPME) method is user-friendly and efficient, requiring minimal plant samples and enabling eco-friendly reuse [23]. Since each method varies in approach and operation, it influences the yield and the physical and chemical properties of the oil. This study extracted EO from *S. fruticosa* aerial parts grown in Egypt using HD, SF, and HS-SPME to assess how the technique impacts yield and volatility of components. In Egypt, HD is the prevalent extraction method for *S. fruticosa* EO [25–28], but this is the first comparison involving EO obtained *via* HS and SF techniques. Results indicated that the extraction method significantly affected the yield, with 0.6 mL/100 g from HD and 0.4 mL/100 g from SF. The observed colors were shiny yellow for HD and dark yellow for SF, Because HS-SPME directly captures volatile constituents, yield measurement is not applicable. Findings show that the composition of the EO is affected both in terms of quality and quantity by the extraction method used for *S*. *fruticosa.* A total of 24, 17, and 19 compounds were identified in the cases of HD, HS, and SF, respectively, accounting for 94.07% (HD), 94.11% (HS), and 69.23% (SF) (Table 1, Figs. S1-3). Variations in the amount and type of identified compounds were evident. In HD EO, eucalyptol (43.87%) and camphor (9.99%) showed the highest percentages, while in HS, as in HD EO, eucalyptol (35.67%) was the most abundant, along with α-thujene (24.88%), (-)-α-pinene (9.66%), and camphene (8.77%). Moreover, in SF, *iso-*caryophyllene (17.07%) and 13-*epi-*manoyl oxide (12.52%) were the main components, whereas eucalyptol accounted for a lower amount than in the HD and HS EOs (7.57%). Additionally, the extraction method affects the distribution of chemical classes, as reflected by differences in *S. fruticosa* EO. Oxygenated components account for the highest percentages in HD and SF EOs, at 68.74% and 41.71%, respectively (Table 2). In the case of HS EOs, non-oxygenated compounds account for the highest percentage at 53.64%, compared to 40.47% for oxygenated components. Furthermore, notable variations were observed among chemical classes, with oxygenated monoterpenes (OM) constituting the most significant percentage in HD EOs at 66.80%, followed by HS at 40.47%, while SF showed the lowest percentage of OM (18.6). In contrast, monoterpene hydrocarbons (MH) form the majority in HS at 51.83%. Moreover, sesquiterpene hydrocarbons (SH) represent the highest percentage in SF EOs, at 26.91%.

**Table 1:** Identified chemical composition of *S. fruticosa* aerial parts EO extracted by HD, HS, and SF

Peak	Rt	Compound	M.F.	RI_exp_		RI_st_	Content %			Ident
							HD	HS	SF	
1	7.325	*α*-Thujene	C_10_H_16_	915	915		5.45	24.88	----	MS, RI
2	7.786	Camphene	C_10_H_16_	935	935		2.15	8.77	----	MS, RI
3	8.685	(-)-*α*-Pinene	C_10_H_16_	965	963.45		2.19	9.66	0.19	MS, RI
4	9.190	Sabinene	C_10_H_16_	983	983.56		2.12	7.92	----	MS, RI
5	9.495	*α*-Phellandrene	C_10_H_16_	994	994		----	0.25	----	MS, RI
6	9.970	(+)-4-Carene	C₁₀H₁₆	1003	998		0.22	----	----	MS, RI
7	10.085	*m*-Cymene	C₁₀H₁₄	1006	1010		1.85	----	----	MS, RI
8	10.361	Eucalyptol	C₁₀H₁₈O	1014	1014		43.87	35.67	7.57	MS, RI
9	11.135	*γ*-Terpinene	C_10_H_16_	1048	1048		0.16	0.35	----	MS, RI
10	11.250	Sabinene hydrate	C_10_H_18_O	1051	1051		----	0.03	----	MS, RI
11	12.220	*β*-Thujone	C₁₀H₁₆O	1083	1081		1.20	0.56	----	MS, RI
12	13.370	Camphor	C_10_H_16_O	1111	1111		9.99	3.28	4.20	MS, RI
13	13.755	*trans*-3-Pinanone	C_10_H_16_O	1132	1134		----	0.25	----	MS, RI
14	13.880	(*1R*)-(+)-Nopinone	C₉H₁₄O	1128	1129		0.75	----	----	MS, RI
15	14.155	*Endo*-Borneol	C_10_H_18_O	1145	1145		----	0.41	1.87	MS, RI
16	14.305	*Iso* borneol	C₁₀H₁₈O	1142	1142		3.20	----	----	MS, RI
17	14.705	Terpinen-4-ol	C_10_H_18_O	1155	1155		0.86	0.09	----	MS, RI
18	15.085	*α*-Terpineol	C₁₀H₁₈O	1167	1167		4.71	----	2.97	MS, RI
19	15.245	Myrtenol	C₁₀H₁₆O	1172	1172		0.43	----	----	MS, RI
20	17.815	(-)-Bornyl acetate	C_12_H_20_O_2_	1259	1259		0.83	----	0.55	MS, RI
21	19.717	*α*-Terpinyl acetate	C_12_H_20_O_2_	1325	1324		0.96	0.18	1.48	MS, RI
22	21.875	*Iso* caryophyllene	C_15_H_24_	1404	1404		----	----	17.07	MS, RI
23	21,915	Caryophyllene	C₁₅H₂₄	1405	1405		3.46	1.43	----	MS, RI
24	22.509	*α*-Guaiene	C_15_H_24_	1427	1428		0.42	0.21	5.08	MS, RI
25	22.832	*α*-Humulene	C_15_H_24_	1436	1436		0.63	0.17	3.23	MS, RI
26	23.810	(+)-Ledene	C_15_H_24_	1478	1474		----	----	1.19	MS, RI
27	24.205	γ-Muurolene	C_15_H_24_	1493	1494		----	----	0.34	MS, RI
28	25.610	(+)- Spathulenol	C_15_H_24_O	1562	1562		0.30	----	1.52	MS, RI
29	25.855	(-)-Globulol	C_15_H_26_O	1575	1576		----	----	0.31	MS, RI
30	25.872	Caryophyllene oxide	C_15_H_24_O	1568	1568		0.41	----	2.44	MS, RI
31	26.218	Viridiflorol	C_15_H_26_O	1584	1584		1.23	----	5.62	MS, RI
32	26.335	Humulene epoxide II	C_15_H_24_O	1599	1599		----	----	0.66	MS, RI
33	34.410	*n*-Hexadecanoic acid	C₁₆H₃₂O₂	1943	1943		6.68	----	----	MS, RI
34	35.940	Dehydroabietan	C_20_H_30_	2020	2021		----	----	0.42	MS, RI
35	36.065	13-*epi*-Manoyl oxide	C_20_H_34_O	2026	2000		----	----	12.52	MS, RI
Total identified compounds							94.07	94.11	69.23	

* *HD* hydrodistillation, *HS* headspace, *SFE* Supercritical fluid extraction

**Table 2:** Percentage of different EOs classes for *S. fruticosa* aerial parts EO extracted by HD, HS, and SF*

Class of compounds	Content %		
	HD	HS	SF
Non-oxygenated compounds	18.65	53.64	27.52
Monoterpene hydrocarbons (MH)	12.29	51.83	0.19
Sesquiterpene hydrocarbons (SH)	4.51	1.81	26.91
Diterpene hydrocarbons (DH)	-----	**-----**	0.42
Aromatic hydrocarbons (ArH)	1.85	**-----**	**-----**
Oxygenated compounds	68.74	40.47	41.71
Oxygenated monoterpenes (OM)	66.80	40.47	18.64
Oxygenated sesquiterpenes (OS)	1.94	**-----**	10.55
Oxygenated diterpenes (OD)	**-----**	**-----**	12.52
Miscellaneous compounds	6.68	**-----**	**-----**

* *HD* hydrodistillation, *HS* headspace, *SFE* Supercritical fluid extraction

### Antibacterial activity

It was tested against six common GIT pathogens, including four Gram-positive bacteria (*C*. *perfringens*, *L*. *monocytogenes*, *S*. *aureus*, and *E*. *faecalis)* and two Gram-negative bacteria (*E*. *coli* and *S. enterica*). The assessment involved measuring zones of inhibition (ZOI), Minimum inhibitory concentration (MICs), Minimum bactericidal concentration (MBCs), time-kill kinetics, and evaluating biofilm inhibition using the crystal violet test.

### Susceptibility test

The antibacterial activity of HD and SF was first assessed using an agar well diffusion assay. against six bacterial strains, with the results presented as ZOI (mm). As shown in Table 3, L. *monocytogenes*, *S. aureus*, and *E. faecalis* demonstrated strong susceptibility to both HD and SF, with ZOI values of 20–30 (20–26), 20–32 (14–30), and 12–27 mm at concentrations of 250–1000 µg/mL of HD and SF, respectively. Additionally, a ZOI of 13–21 mm was observed against *E. faecalis* at 500–1000 µg/mL of SF. This effect was more significant than that of the standard tested antibiotics, which had ZOI values of 7–8 and 21–25 mm.

Following this, *C. perfringens* and *S. enterica* showed moderate susceptibility to SF, especially at the higher concentration of 1000 µg/mL, with a ZOI value of 12 mm, which is relatively lower than the standard antibiotics’ range of 18–35 mm. Notably, these strains exhibited resistance to HD. Additionally, *E. coli* demonstrated resistance to both HD and SF. Low ZOI measurements may be due to the oils not diffusing well in the agar medium. To get a more accurate picture of the oils’ inhibitory effects, we measured MICs and MBCs as part of a follow-up investigation.

**Table 3:** Zone of inhibition (ZOI) of the *S. fruticosa* aerial parts extracted essential oils by HD and SF against tested reference strains

**Reference strains**	**ZOI (mm) **								
	**Sample conc. µg/mL /**						**AK 30**	**AX 25**	**NOR 10**
	**HD**			**SFE**					
	**1000**	**500**	**250**	**1000**	**500**	**250**			
*C. perfringens *ATCC 13124	-	-	-	12	-	-	23	18	34
*L. monocytogenes *ATCC 7644	30	20	20	26	24	20	7	7	8
*S. aureus *ATCC 25923	32	22	20	30	20	14	22	21	25
*E. faecalis *ATCC 29212	27	14	12	21	13	-	22	21	25
E. coli ATCC 8739	-	-	-	-	-	-	22	-	30
S. enterica ATCC 14028	-	-	-	12	11	10	21	21	35

Results were expressed as mean (*n* = 3) with SD ± 1. (-), not detected; (ZOI), zone of inhibition measured in mm; (AK 30), amikacin; (AX 25), amoxicillin; (NOR 10), norfloxacin.

### Measurements of MIC and MBC

As shown in Tables 4 and 5, and 6, the MIC results and corresponding viability percentages reveal that the investigated HD and SF have a promising ability to inhibit the growth of specific bacterial strains that cause GT infection. The potency levels of these strains vary. Notably, the SF showed more potent activity than the HD; it demonstrated strong growth inhibition against the tested Gram-positive bacteria with MICs of 3.91 and 125 µg/mL, and against the Gram-positive *E*. *faecalis* with an MIC of 15.63 µg/mL, with the lowest viability percentage of 6.08% against *S. aureus* at 1.95 µg/mL. Meanwhile, the HD showed inhibitory activity against the tested Gram-negative bacteria *E. coli* and *S. enterica*, with MICs of 62.5 and 125 µg/mL, respectively, which are lower than those of SF (MICs = 250 µg/mL), with the lowest viability percentage of 5.53% against *S. enterica* at 62.5 µg/mL. This effect was more significant than that of the standard tested antibiotics, which had MICs ranging from 125 to 500 µg/mL. For the tested EO_S_, the MBC values were generally higher than the MICs, with significant variations among the different strains. Table 4 shows the MBC values (µg/mL) recorded, which range from 31.25 to 500 µg/mL for both HD and SF. Their low MIC and MBC values demonstrate the strong effectiveness of the EOs. The bactericidal effect of the tested EOs was further verified through the time-kill kinetics analysis.

**Table 4:** MICs and MBCs of the *S. fruticosa* aerial parts extracted essential oils by HD and SF methods

Reference strains	MIC µg/mL				MBC µg/mL	
	HD	SFE	C	CIP	HD	SFE
*C. perfringens* ATCC 13124	250	125	500	500	500	500
*L. monocytogenes* ATCC 7644	62.5	3.91	500	250	125	62.5
*S. aureus* ATCC 25923	31.25	3.91	250	500	31.25	31.25
*E. faecalis* ATCC 29212	62.5	15.63	250	125	125	62.5
*E. coli* ATCC 8739	62.5	250	500	250	500	500
*S. enterica* ATCC 14028	125	250	250	250	250	500

*MIC* minimum inhibitory concentration, *MBC* minimum bactericidal concentration. Results were expressed as mean (*n* = 3); (CIP, 1.0 mg/mL), ciprofloxacin; (C, 1.0 mg/mL), chloramphenicol

**Table 5:** Dose–response effect of the *S. fruticosa* aerial parts extracted essential oils by the HD method, on the viability % of the GT pathogens in the broth dilution assay

Samples	Conc. µg/mL*									
	1000	500	250	125	62.5	31.25	15.63	7.81	3.91	1.95
*C. perfringens* ATCC 13124	0.00 ^a^	0.00 ^a^	0.00 ^a^	7.01 ^b^	11.87 ^c^	17.17 ^d^	32.90 ^e^	47.21 ^f^	55.79 ^g^	60.09 ^h^
*L. monocytogenes* ATCC 7644	0.00 ^a^	0.00 ^a^	0.00 ^a^	0.00 ^a^	0.00 ^a^	8.53 ^b^	24.55 ^c^	33.59 ^d^	40.05 ^e^	55.56 ^f^
*S. aureus* ATCC 25923	0.00 ^a^	0.00 ^a^	0.00 ^a^	0.00 ^a^	0.00 ^a^	0.00 ^a^	12.92 ^b^	25.84 ^c^	42.55 ^d^	56.23 ^e^
*E. faecalis* ATCC 29212	0.00 ^a^	0.00 ^a^	0.00 ^a^	0.00 ^a^	0.00 ^a^	5.81 ^b^	19.37 ^c^	35.77 ^d^	52.16 ^e^	65.57 ^f^
*E. coli* ATCC 8739	0.00 ^a^	0.00 ^a^	0.00 ^a^	0.00 ^a^	0.00 ^a^	10.87 ^b^	24.46 ^c^	38.04 ^d^	57.07 e	63.86 ^f^
*S. enterica* ATCC 14028	0.00 ^a^	0.00 ^a^	0.00 ^a^	0.00 ^a^	5.53 ^**b**^	11.69 ^c^	25.28 ^d^	45.81 ^e^	58.45 ^f^	71.09 ^g^

Different letters of the means are significantly different (*p* < 0.00001) using Duncan’s post hoc test. Results were expressed as mean (*n* = 3)

**Table 6:** Dose–response effect of the *S. fruticosa* aerial parts extracted essential oils by the SF method, on viability % of the GT pathogens in the broth dilution assay

Samples	Conc. µg/mL*									
	1000	500	250	125	62.5	31.25	15.63	7.81	3.91	1.95
*C. perfringens* ATCC 13124	0.00 ^a^	0.00 ^a^	0.00 ^a^	0.00 ^a^	10.01 ^b^	24.32 ^c^	41.49 ^d^	58.66 ^e^	67.24 ^f^	74.39 ^g^
*L. monocytogenes* ATCC 7644	0.00 ^a^	0.00^a^	0.00 ^a^	0.00 ^a^	0.00 ^a^	0.00 ^a^	0.00 ^a^	0.00 ^a^	0.00 ^a^	10.34 ^b^
*S. aureus* ATCC 25923	0.00 ^a^	0.00 ^a^	0.00 ^a^	0.00 ^a^	0.00 ^a^	0.00 ^a^	0.00 ^a^	0.00 ^a^	0.00 ^a^	**6.08** ^b^
*E. faecalis* ATCC 29212	0.00 ^a^	0.00 ^a^	0.00 ^a^	0.00 ^a^	0.00 ^a^	0.00 ^a^	0.00 ^a^	11.03 ^b^	22.35 ^c^	41.73 ^d^
*E. coli* ATCC 8739	0.00 ^a^	0.00^a^	0.00 ^a^	9.78 ^b^	33.97^c^	42.12^d^	52.99 ^e^	65.22 ^f^	70.65 ^g^	80.16 ^h^
*S. enterica* ATCC 14028	0.00 ^a^	0.00 ^a^	0.00 ^a^	12.80^b^	23.70 ^c^	44.23^d^	50.55 ^e^	77.41 ^f^	80.57 ^g^	96.37 ^h^

Different letters of the means are significantly different (*p* < 0.00001) using Duncan’s post hoc test. Results were expressed as mean (*n* = 3)

### Time kill assay

**Fig. 1:**
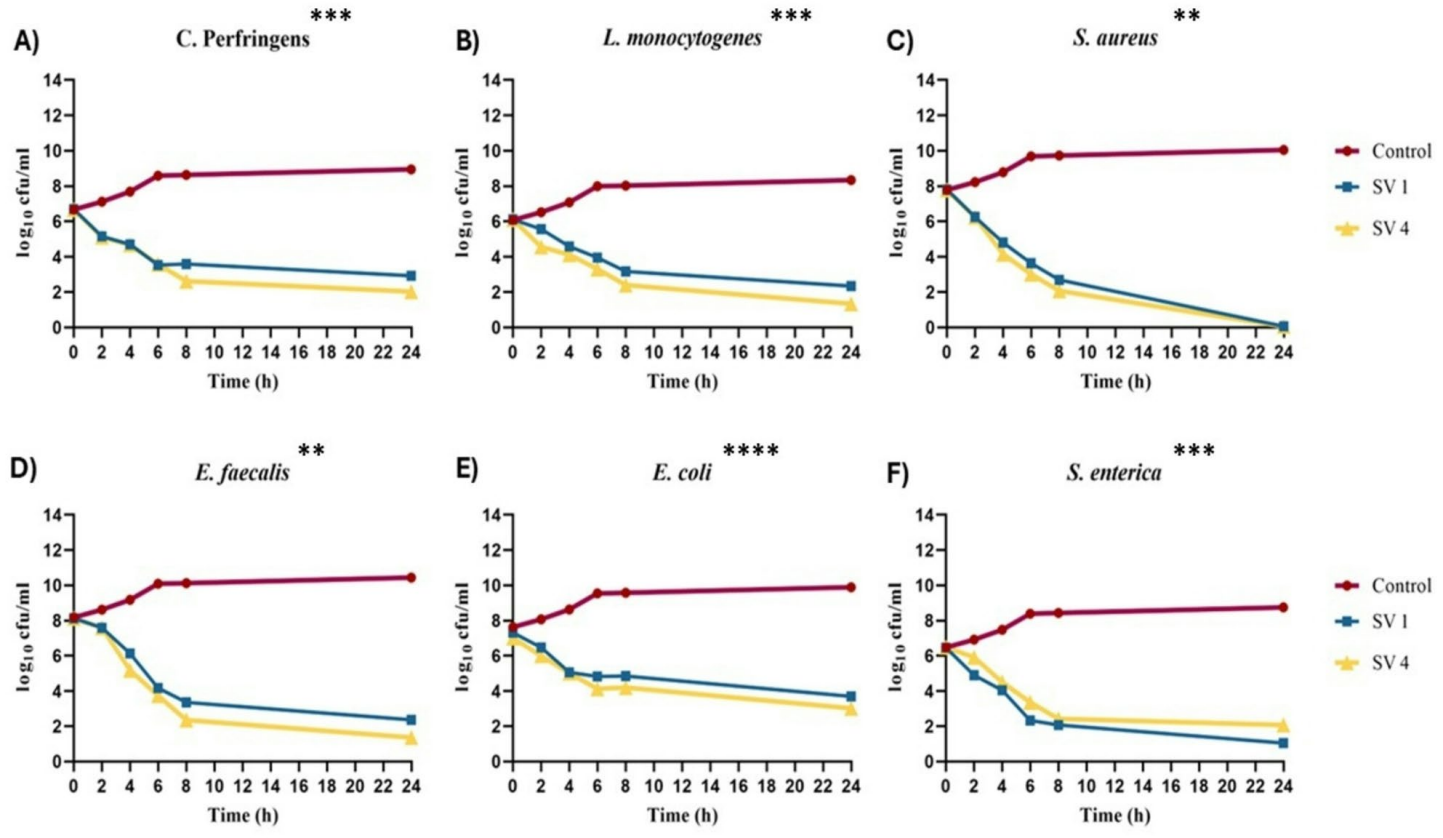
Time kill kinetics of HD and SF at 2× MIC, against the six tested gastrointestinal tract infection pathogens, **A**) *C. perfringens* (*P*-value ***=0.0003); **B**) *L. monocytogenes* (*P*-value ***=0.0004); **C**) *S. aureus* (*P*-value **=0.007); **D**) *E. faecalis* (*P*-value **=0.003); **(E)** *E. coli* (*P*-value ****= ˂ 0.0001); and **(F)** *S. enterica* (*P*-value ***=0.0007). Results were expressed as mean (*n* = 3)

Figure 1 displays the time-kill kinetics of HD and SF-EOs against six bacterial strains. The kinetics of the EOs were comparable to those of the bacterial control. Notably, the bactericidal activity was quite similar after treatment with HD and SF-EOs against *C. perfringens*, *S*. *aureus*, and *E*. *coli.* At first, the effect was only weakly inhibitory, but it eventually became bacteriostatic. Here are the reductions in bacterial colony-forming units (cfu/mL) at various time points: 0 h − 6.7 log_10_ CFU/mL, 2 h − 5.2 log_10_ CFU/mL, 4 h − 4.7 log_10_ CFU/mL, and 6 h − 3.5 log_10_ CFU/mL, indicating bactericidal effects. This bactericidal activity lasted for up to 24 h, with reductions of 2.9, 0.07, and 3.7 log_10_ cfu/mL against *C. perfringens*, *S*. *aureus*, and *E*. *coli*, respectively (Fig. 1A, C, and E).

Notably, the SF EOs showed a greater reduction effect against *L*. *monocytogenes* and *E*. *faecalis* compared to HD. Starting with the bacteriostatic effect 4 h after treatment, both SF and HD caused reductions of 4.1–5.1 and 4.1–6.1 log_10_ CFU/mL, respectively. Next, there was a rise in bactericidal activity, with reductions of 3.9–1.3 (3.9–2.3) log_10_ CFU /mL for *L*. *monocytogenes* and 4.1–1.4 (4.1–2.4) log_10_ CFU /mL for *E. faecalis* after 6 to 24 h of treatment with SF and (HD) (Fig. 1B and D).

Additionally, HD exhibited a greater reduction in *S*. *enterica* count than the SF, with a bacteriostatic effect of 4.4 (4.5) log_10_ CFU /mL after 4 h of incubation, followed by a rise in bactericidal activity of 3.3–1.7 (3.4–2.1) log_10_ CFU /mL after 6–24 h of incubation (Fig. 1F).

### Antibiofilm quantitative assay

**Fig. 2:**
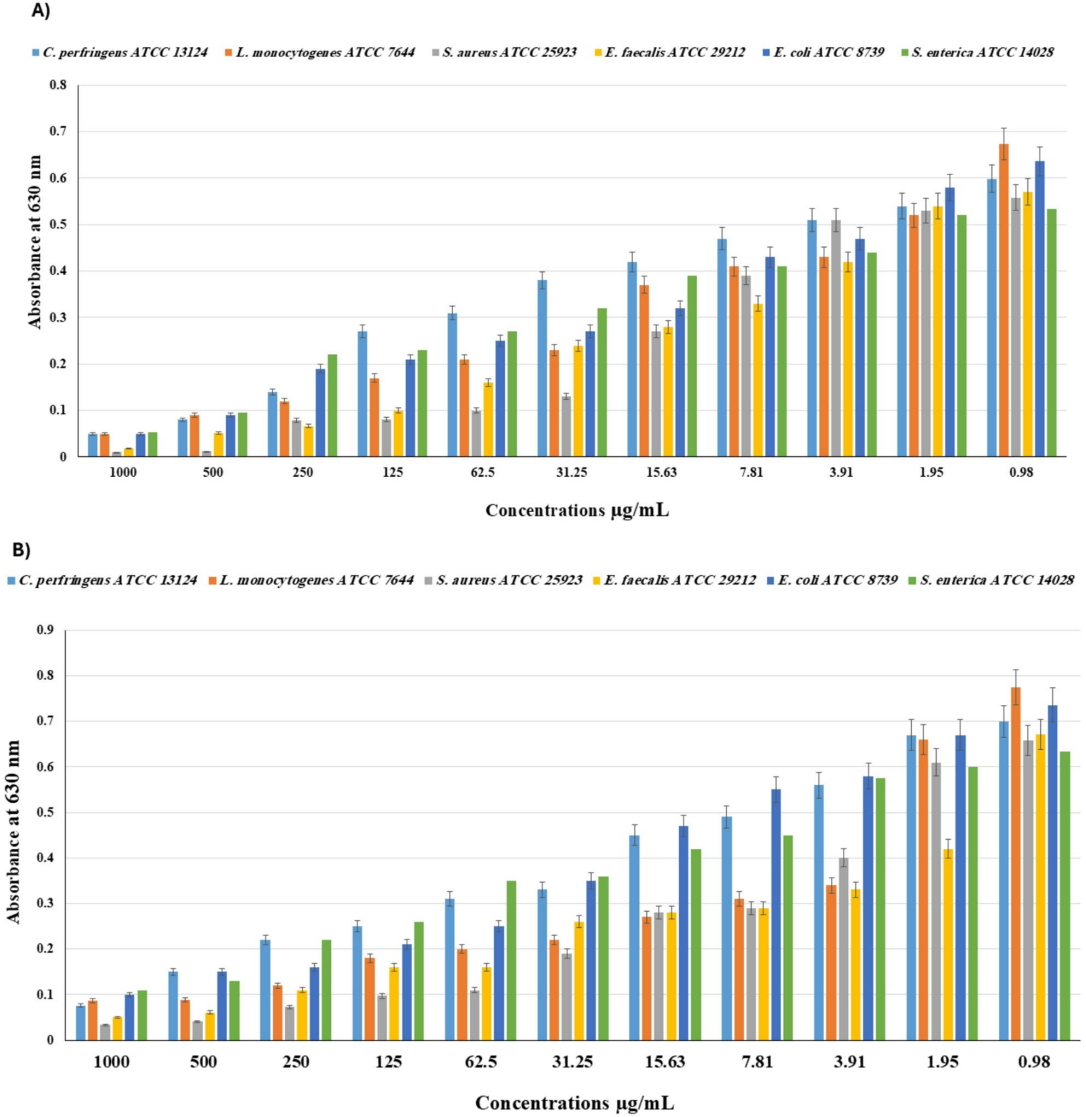
Dose–response effect of HD (**A**); and SF (**B**), on selected Gastrointestinal infection pathogens in biofilm formation test with concentration value “0” as a control. Error bars represent SD from the means (*n* = 3). The results indicate that both extracts demonstrated significant (*p* < 0.000001) biofilm inhibition across all tested concentrations

The biofilm inhibition percentages (BI %) of HD and SF EOs against various bacterial strains are summarized in Tables 7 and 8, and Fig. 2. The results show that both EOs exhibited significant (*p* < 0.000001) biofilm inhibition across all tested concentrations, with the highest concentrations producing the most notable effects. Notably, HD EO shows higher potency against the six bacterial strains at 1000 µg/mL compared to other treatments, SF. For the Gram-positive bacteria, the BI% was 91.65–98.21 (88.89–94.98), which was greater than the values obtained against the Gram-negative bacteria, 89.87–96.85 (82.62–92.55%). As the concentration decreased, the inhibition also reduced. Notably, at the lowest tested concentration of 1.95 µg/mL, the HD exhibited 9.85 and 22.85 BI% against *C*. *perfringens* and *L*. *monocytogenes*, greater than the BI% of 4.15 and 14.73 for SF. In contrast, for *S. aureus*, *E. faecalis*, *E. coli*, and *S. enerica*, the SF exhibited increased BI% of 7.29, 37.41, 8.97, and 5.21, compared to BI% of 5.02, 5.43, 8.81, and 2.44 for the HD.

**Table 7:** Biofilm inhibition % (BI%) of the *S*. *fruticosa* aerial parts’ essential oils extracted by the HD method

Samples	Conc. µg/mL*									
	1000	500	250	125	62.5	31.25	15.63	7.81	3.91	1.95
*C. perfringens* ATCC 13124	91.65	86.64	76.63	54.92	48.25	36.56	29.88	21.54	14.86	9.85
*L. monocytogenes* ATCC 7644	92.58	86.65	82.20	74.78	68.84	65.88	45.10	39.17	36.20	22.85
*S. aureus* ATCC 25923	98.21	97.85	85.84	85.48	82.08	76.70	51.61	30.11	8.60	5.02
*E. faecalis* ATCC 29212	96.85	90.89	88.27	82.49	71.98	57.97	50.96	42.21	26.44	5.43
*E. coli* ATCC 8739	92.14	85.85	70.13	66.98	60.69	57.55	49.69	32.39	26.10	8.81
*S. enterica* ATCC 14028	89.87	82.18	58.72	56.85	49.34	39.96	26.83	23.08	17.45	2.44

*P-value* * < 0.000001. Results were expressed as mean (*n* = 3)

**Table 8:** Biofilm inhibition % (BI%) of the *S. fruticosa* aerial parts’ essential oils extracted by the SF method

Samples	Conc. µg/mL*									
	1000	500	250	125	62.5	31.25	15.63	7.81	3.91	1.95
*C. perfringens* ATCC 13124	89.13	78.54	68.53	64.23	55.65	52.79	35.62	29.90	19.89	4.15
*L. monocytogenes* ATCC 7644	88.89	88.50	84.50	76.74	74.16	71.58	65.12	59.95	56.07	14.73
*S. aureus* ATCC 25923	94.98	93.77	88.91	85.26	83.28	71.12	57.45	55.93	39.21	7.29
*E. faecalis* ATCC 29212	92.55	90.91	83.61	76.15	76.15	61.25	58.27	56.78	50.82	37.41
*E. coli* ATCC 8739	86.41	79.62	78.26	71.47	66.03	52.45	36.14	25.27	21.20	8.97
*S. enterica* ATCC 14028	82.62	79.46	65.24	58.93	44.71	43.13	33.65	28.91	9.16	5.21

*P-value* * < 0.000001. Results were expressed as mean (*n* = 3)

**Figure:**
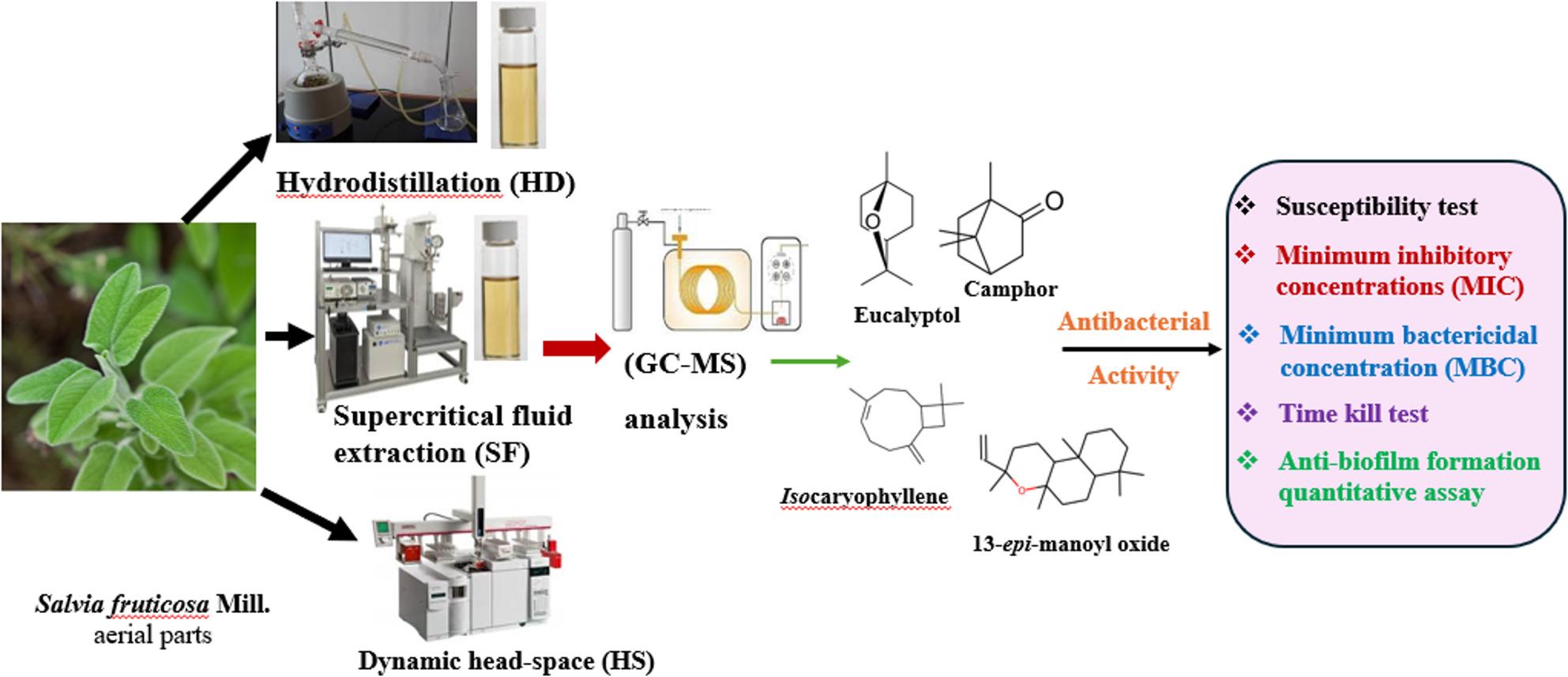
Experimental design

## Discussion

The EO of *S. fruticosa* was obtained using three methods to evaluate how different extraction techniques affect the physical and chemical properties of the EOs. The SF technique produced higher EO output than HD because supercritical CO2 (SCC) was used as the extraction solvent. SCC’s low viscosity and surface tension improve penetration, leading to more effective extraction and higher oil yield [47]. Its solvation power also co-solubilizes fatty components, giving SF oils a dark color and viscous consistency. The extracted EOs were analyzed by GC/MS for qualitative and quantitative identification. The results showed that volatile components varied by extraction method, likely due to changes in plant sample condition, pressure, temperature, and extraction time. In SF, dried plant material was extracted with SCC; in HD, fresh material was boiled in water; and in HS, the fresh sample was heated until the essential oil volatilized, explaining the observed differences [38, 51]. Although both HD and HS used fresh materials, temperature and time significantly affected the stability and quantity of volatile components. The solvent choice is also critical. In HD, water vapor penetrates better, extracting low-molecular-weight oxygenated and non-oxygenated compounds more than high-molecular-weight ones, due to longer times and higher temperatures [52, 53]. SF uses SCC, a lipophilic solvent with broad selectivity, detecting 13-epi-manoyl oxide only in SF-EOs. HS is a cutting-edge technique that extracts volatile components with different boiling points without artifacts [54]. The present findings align with previous reports on *S. fruticosa*.

EO extracted by HD from different Egyptian regions [25–28], showing that oxygenated monoterpenes are the main components (Eucalyptol, 43.87%). Variations in components likely result from environmental conditions, genetics, collection timing, drying, temperature, and plant age, which significantly affect their chemical makeup [55, 56].

The antibacterial activity of the extracted EOs was tested against six GIT pathogens: *C*. *perfringens*, *L*. *monocytogenes*, *S.* aureus, *E*. *faecalis*, *E*. *coli*, and *S. enterica*, selected for their availability and association with GIT infections. *C*. *perfringens*, a Gram-positive, anaerobic, spore-forming bacterium, is a common foodborne pathogen and gut commensal that causes diarrheal illnesses and enteric diseases through toxins [57–58]. It also forms spores and biofilms, aiding environmental persistence and transmission [59].

*L. monocytogenes* is a foodborne pathogen causing human listeriosis, prevalent in water, soil, and vegetation [60]. It usually leads to gastroenteritis, but can cause severe invasive disease in immunocompromised people. Symptoms include fever, diarrhea, nausea, headache, and joint and muscle pains [61]. *S. aureus* is a major pathogen causing infections across organs in hospitals and communities, leading to diseases like antibiotic-associated diarrhea [62]. Reduced stomach acidity aids colonization by helping it survive gastric barriers [62]. Gut overgrowth causes enteritis and diarrhea and is linked to inflammatory bowel disease through gut antigen-triggered inflammation [63]. Its toxins damage intestinal barriers by disrupting cell junctions [64]. *E*. *faecalis* is a Gram-positive bacterium common in the human GIT and an opportunistic pathogen of growing concern. It is among the first colonizers of the infant GIT [65]. Usually harmless, disrupting its relationship with the host can make it pathogenic, causing life-threatening infections like bacteremia and intra-abdominal infections, often with high mortality [66]. *E. coli* is the most common aerobic species in the human gut. Normal strains are commensals that interact positively with the microbiota and support the host by providing essential nutrients, such as vitamins. Diet mainly influences gut microbiota structure [67]. Pathogenic *E*. *coli* causes severe diarrhea via enterotoxins and other damaging toxins [68], worsening inflammatory bowel disease [69]. It can also cause vomiting, bloody diarrhea, hemorrhagic colitis, and hemolytic uremic syndrome.

Salmonella infections caused by various serotypes, such as *S*. *enterica*, pose significant economic and public health issues. Salmonellosis manifests as gastroenteritis, inflammation, enteric fever, bacteremia, and other conditions [70]. The most common form, *Salmonella gastroenteritis*, causes stomach cramps, diarrhea, fever, and sometimes vomiting [71]. Human cases often result from the ingestion of contaminated food, enabling Salmonella to colonize the gut [71]. Four tests evaluated the antibacterial effects of the EOs: agar diffusion, broth microdilution, time-kill assay, and biofilm formation. Results showed *L*. *monocytogenes*, *S. aureus*, and *E*. *faecalis* were highly susceptible to HD and SF, while *E. coli* and *S. enterica* were resistant to both oils. This suggests that Gram-positive strains are more sensitive to EOs in a dose-dependent manner, while Gram-negative bacteria are less sensitive, likely due to their peptidoglycan layer outside the outer membrane. Gram-negative bacteria have a double phospholipid membrane with lipopolysaccharides, which block hydrophobic EO components and enable rapid resistance [72].

While the agar diffusion test shows that antibacterial agents spread quickly through solid media [43], this can sometimes lead to deviations due to variability in volatile compound diffusion. To improve accuracy and address agar diffusion limitations [41, 43], we used a microdilution test. Results showed SF-EOs strongly inhibited gram-positive bacteria in a dose-dependent manner, while HD-EOs inhibited gram-negative bacteria. Thus, the promising antibacterial activity of SF-EOs against gram-positive bacteria and HD-EOs against gram-negative bacteria is not detectable by the agar diffusion test. The oils’ antibacterial effects are linked to their chemical compounds. For example, the HD-EOs of *S*. *fruticosa* are rich in oxygenated monoterpenes, such as eucalyptol, and exhibit promising antimicrobial activity [73–76]. Camphor also has strong antibacterial properties [77, 78]. SF-EOs contain a high percentage of *iso-c*aryophyllene (17.07%, *β*-caryophyllene), which is effective against Gram-positive bacteria [79–81]. It contains 13-*epi*-manoyl oxide, which shows stronger antibacterial activity against Gram-positive bacteria [82]. The activity may result from main compounds and their interactions with each other and other compounds [83, 84]. The combined effects of EO compounds may increase plasma membrane permeability, consistent with reports that EO components act synergistically and that their biosynthesis and accumulation influence biological activity [85].

It has been reported that eucalyptol, abundant in HD-EOs, shows potent antibacterial effects against *S*. *aureus*. This effect alters the bacterial surface charge and triggers oxidative stress, leading to membrane damage, leakage of internal contents, and lipid oxidation. Additionally, ROS buildup hampers antioxidant enzymes and harms large molecules, leading to bacterial damage and cell death [86]. Additionally, it can influence the carbohydrate metabolism of *S. enterica* [87].

The time–kill test is a reliable method for assessing the bactericidal potential of an agent [43]. As illustrated, both EOs demonstrated kinetics comparable to those of the bacterial control, indicating their potential effectiveness as antimicrobial agents. Of great significance, the HD and SF-EOs demonstrated bactericidal properties leading to a decline of 3.8 (4.68), 3.94 (4.94), 7.76 (7.85), 6.2 (7.19), 3.7 (4), and 5.47 (4.46) log_10_ CFU/mL, respectively, in bacterial count of the tested GIT pathogens: *C. perfringens*, *L. monocytogenes*, *S. aureus E. faecalis*, *E. coli*, and *S. enterica* compared to the initial inoculum, and persisted for 24 h of treatment at concentration of 2x MIC.

The antimicrobial results from this study show that *S*. *fruticose* EO, commonly found in Egypt, exhibits strong activity, with MIC values of 31.25–250 µg/mL for HD and 3.91–250 µg/mL for SF-EOs. These results are better than those reported by Giweli et al. [32], who found that *S. fruticosa* essential oil had MIC values of 0.125-1.5 mg/mL and MBC values of 0.5-2.0 mg/mL. Biofilms differ significantly from suspended bacteria because they are in their natural habitat. Research shows EOs can prevent biofilm formation in several ways. They may disrupt the cytoplasmic membrane, causing leakage of contents or enzyme inactivation [88]; or interfere with quorum sensing, suppressing flagellar gene transcription and motility [89]; reduce bacterial adherence to surfaces [90]; or induce reactive oxygen species buildup, leading to oxidative stress and cell death [91]. This study focused on EO’s ability to inhibit biofilm development. Results showed that both oils inhibited Gram-positive bacterial biofilms more effectively than Gram-negative biofilms. HD-EOs were more potent at high doses against six strains than SF-EOs. HD-EOs, rich in eucalyptol, strongly inhibit *E. coli* [73] and *S. aureus* [92]. We hypothesize that each EO’s unique makeup might limit resistance, unlike synthetic agents [93]. While some EOs are safe, more research is needed on their safety and dosing in clinical use.

## Conclusion

EOs from *S*. *fruticosa* aerial parts were extracted using three methods, and their compositions were qualitatively and quantitatively compared. The SF technique produced a higher EOs yield. Eucalyptol was the dominant compound in both HD and HS-EOs, while *iso-*caryophyllene and 13-*epi*-manoyl oxide were the main constituents of SF-EO. Both HD and SF-EOs were active against *L*. *monocytogenes*,* S*. *aureus*, and *E. faecalis*; additionally, SF-EO affected *S*. *enterica* in agar diffusion tests. SF-EO showed strong inhibition of *L*. *monocytogenes*,* S*. *aureus*, and *E. faecalis* growth, surpassing HD-EO in microdilution assays. HD-EO is mainly effective against *E*. *coli.* Both oils significantly inhibited biofilm formation in Gram-positive bacteria more than in Gram-negative bacteria. The volatile compounds in *S*. *fruticosa* exhibit antibacterial activity against gastrointestinal pathogens, suggesting their potential as bioactive agents for the treatment of GIT infections. Nonetheless, further studies on toxicity, including in vivo tests and pharmacokinetics, are necessary to confirm their safety for clinical use.

## Supplementary Information

Supplementary Material 1.

## Data Availability

The datasets used and/or analyzed during the current study are available from the corresponding author on responsible request.

## Abbreviations

EOs: Essential Oils
HD: Hydrodistillation
SF: Supercritical Fluid
HS: Head- Space
MBC: Minimum Bactericidal Concentration
CFU: Colony-forming Unit
MIC: Minimum Inhibitory Concentration
SPME: Solid-phase Microextraction
GIT: Gastrointestinal Tract
GC/MS: Gas Chromatography-Mass Spectrometry
MHA: Mueller Hinton Agar
MHB: Mueller Hinton Broth
DMSO: Dimethyl Sulfoxide
AK: Amikacin
AX: Amoxycillin
NOR: Norfloxacin
ZOI: Zone of Inhibition
BI%: Biofilm Inhibition Percentages
SCC: Supercritical Carbon Dioxide
APCI: Atmospheric Pressure Chemical Ionization
CLSI: Clinical and Laboratory Standards Institute
