# Supplementary material for: Impact of extraction method on the chemical composition and antibacterial potential of Salvia fruticosa essential oil
Source: BMC Complement Med Ther. 2026 May 2;26:165. doi: 10.1186/s12906-026-05376-5 (PMC13135703; doi:10.1186/s12906-026-05376-5)
Supplement: Supplementary file 1 — Supplementary Material 1. [file 12906_2026_5376_MOESM1_ESM.docx]

**Essential Oils Extraction**

***Hydrodistillation (HD)***

We chopped the fresh aerial parts into small pieces (250 g) and blended them with distilled water. The mixture was then hydrodistilled for four hours using a Clevenger distillation apparatus [38]. The oil obtained through HD was dried using anhydrous sodium sulfate (Sigma-Aldrich, USA).

***Supercritical fluid extraction (SF)***

It was performed using supercritical carbon dioxide with a Speed TM SFE-2/4, and functional separations were carried out using the USDA1-USA. 160 g of air-dried samples were extracted at 40°C and 100 bar with a flow rate of 10 mL/min. First, the device was operated in static mode for one hour, followed by two hours of dynamic mode, for a total of three hours. To address the low polarity and thus the reduced effectiveness of CO_2_ in extracting more polar compounds from natural matrices, absolute ethanol was added as a cosolvent to adjust polarity and improve solvating power (flow rate of 2 mL/min) [99, 38]. The EOs percentages obtained from HD and SF-EOs were reported in mL/100 g of aerial parts. [38].

***Dynamic head-space GC/MS analysis***

Hashemi et al. [39] followed a specific procedure. Approximately 2 g of aerial parts was placed in a glass vial (5 mL). A Shimadzu headspace sampler HS-20, connected to a Shimadzu GC-MS-QP2020 from Kyoto, Japan, was used. The setup included an Rtx-1MS column, 30 m long, 0.25 mm ID, and 0.25 µm film thickness (Restek, Bellefonte, PA, USA). The oven temperature was set at 80 °C, while the sample and transfer line temperatures were set at 150 °C. The condition of headspace sampling included an 8-minute equilibration period, a 2-minute pressurization phase, and a 5-minute needle flush. The column oven temperature was initially set at 45 °C for 2 minutes, then ramped up to 300 °C at a rate of 5 °C per minute and maintained for 5 minutes. Helium was used as the carrier gas at a flow rate of 1.40 mL/min, with a split ratio of 1:25. The Atmospheric Pressure Chemical Ionization (APCI) pressure was kept steady at 50 kPa, while the ion source and interface temperatures were set at 200 °C and 280 °C, respectively

**Susceptibility test**

Using the agar well-diffusion method, we performed the assay according to guidelines from the Clinical and Laboratory Standards Institute CLSI, 2025[40] and Gholizadeh et al. [41]. We tested four Gram-positive bacteria (*C*. *perfringens* ATCC 13124, *L*. *monocytogenes* ATCC 7644, *S*. *aureus* ATCC 25923, and *E.* *faecalis* ATCC 29212) and two Gram-negative bacteria (*E*. *coli* ATCC 8739 and *S*. *enterica* ATCC 14028). As reference drugs, we used conventional antibiotics with various mechanisms of action (Bioanalyse, Turkey): Amikacin (AK 30) as a protein synthesis inhibitor, amoxicillin (AX 25) as a cell wall synthesis inhibitor, and norfloxacin (NOR 10) as a DNA replication inhibitor while 10% Dimethyl sulfoxide (DMSO) as a negative control. We prepared a 100 µL suspension containing 1××10^5^ cells of each reference strain (optical density, OD600 = 0.2) and inoculated it onto MHA plates. After the agar solidified, we drilled wells with a diameter of 0.6 cm and added 50 µL of the tested sample at concentrations of 50, 1, and 0.5 mg/mL. We then refrigerated the plates to facilitate diffusion of the tested samples [42] and incubated them at 37°C for 24 hours. For *C*. *perfringens*, we incubated anaerobically using an anaerobic jar. We measured the antimicrobial activity of the extracts by the diameter of the zones of inhibition (ZOI) in millimeters.

**Determination of minimum inhibitory concentrations (MIC)**

For HD and SF, the assay was conducted using a broth microdilution method, following the guidelines of Balouiri et al. [43] and CLSI, 2025[40]. We prepared stock solutions by dissolving 50 mg of each oil separately in 1 mL of 10% DMSO. Each well (2–12) was given 100 μL of sterile MHB, and 150 μL of the stock solution was added to the first column of microtiter plates. A two-fold serial dilution of the stock solution was made by transferring 100 μL from the first well to the next wells, up to the 11th. We then added 50 μL of bacterial inoculum containing 1 × 105 CFU/mL (OD600 = 0.2) to each well, except the last (blank). All plates were then incubated for 24 hours at 37°C, except for *C.* *perfringens,* which was incubated in an anaerobic jar. We used chloramphenicol (1 mg/mL) and ciprofloxacin (1 mg/mL) as standards. We measured absorbance at λmax = 620 nm using an automated microplate reader (ChroMate 4300, USA). We determined statistical significance with a *p*-value of < 0.05 compared to the control. We calculated the cell viability percentage using the formula (A _treated_/A /A _untreated_) × 100. A is the absorbance at 620 nm, with the viability of untreated cells set at 100%.

**Assessment of the minimum bactericidal concentration (MBC)**

Using the broth dilution method, as outlined in the CLSI, 2025 [40] guidelines, we determined the results. We took 50 µL aliquots from all wells without visible growth after 24 hours of incubation at 37 °C and spread them onto MHA plates. These plates were then incubated for another 24 hours at 37°C, except for *C. perfringens*, which was incubated in an anaerobic environment. Since the detection limit (LOD) for this method is 10 CFU/mL, no growth on the
MHA plates indicated that the bacterial concentration was below this level. This confirmed that the initial concentration of 105 CFU/mL had been reduced to less than 10 CFU/mL. Each test was repeated three times to ensure consistent results.

**Time kills assay**

Following the methods outlined by Foerster et al. [44], we prepared a 1 × 10^5 CFU/mL concentration of each tested strain in 50 mL MHB separately through incubation at 37 °C with shaking at 150 rpm for four hours. We then added 5 mL of each sample (2× MIC) to each flask containing the pre-incubated bacteria. At various time points (0, 2, 4, 6, 8, and 24 hours) at 37 °C, we took aliquots and serially diluted them. We plated 100 microliters of each dilution on MHA and incubated it at 37 °C for 24 hours, including both positive and negative controls with inoculated and uninoculated MHB. During the initial 6-hour time-kill experiments, we monitored bacterial growth by measuring changes in viable bacteria (CFU/mL). We analyzed the data by calculating the average colony counts (Log10 CFU/mL) from three replicates per dilution at each time point. We set the detection limit at 2 Log10 CFU/mL. We defined bacteriostatic and bactericidal effects as reductions of 2 and 3 Log10 CFU/mL, respectively, compared to the initial inoculum.

***Anti-biofilm formation quantitative assay***

Our experiment followed the methods outlined by Kang et al. [46] and Salem et al. [47], with some minor changes. In brief, we added different concentrations of HD and SF oils (0.097-50 mg/mL) to 96-well polystyrene plates and inoculated each plate with 100 μL of a microbial culture (1 × 10^5 CFU/mL) individually. The plates were then incubated statically for 48 hours at 37°C, with anaerobic conditions for *C.* *perfringens*. After incubation, we washed the plates twice with sterile PBS (pH 7.3) and air-dried them. To fix the biofilms, we added 400 μL of methanol (MeOH) for 15 minutes, followed by staining with a 1% crystal violet solution for 10 minutes. We then solubilized the stained biofilms by adding absolute ethanol to each well. We measured the absorbance at 630 nm using a microplate reader (ChroMate 4300, USA). The results were expressed as the percentage of biofilm formation inhibition, calculated using the formula: [(OD control – OD sample) / (OD control)] × 100, where OD represents optical density at 630 nm, and untreated cells served as the control.

**
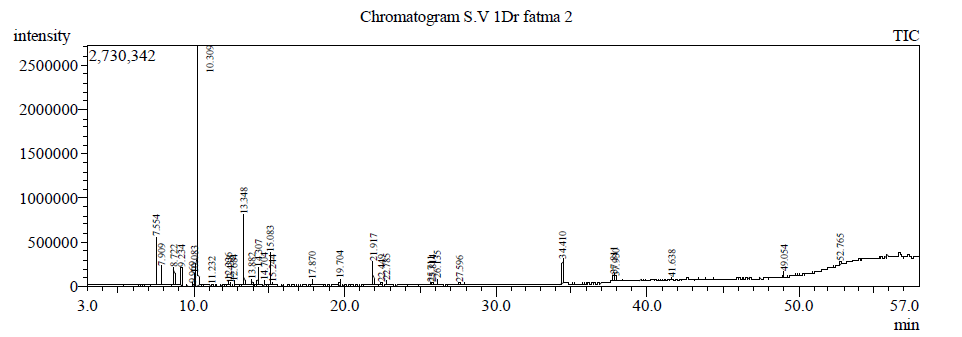
**

**Fig. S1** GC/MS chromatogram of essential oil extracted by HD from *S. fruticosa* aerial parts.

**
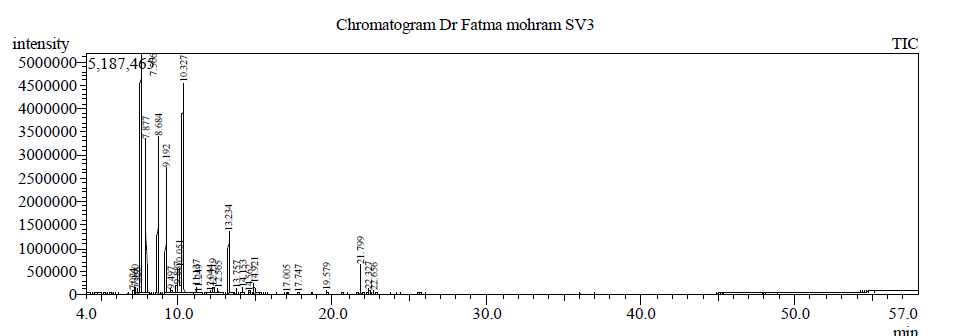
**

**Fig. S2** GC/MS-HS chromatogram of essential oil from *S. fruticosa* aerial parts.

**
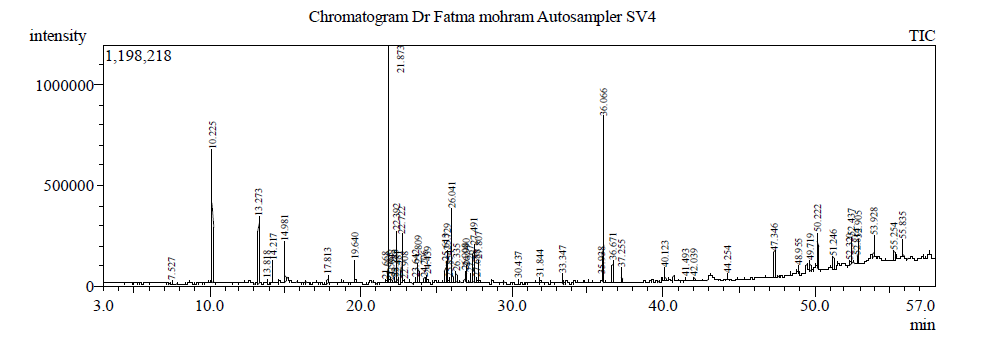
**

**Fig. S3** GC/MS chromatogram of essential oil extracted by SF from *S. fruticosa* aerial parts.

**References**

Jati PTS, Wiradiestia D, Altway A, Winardi S, Machmudah S. Extraction process optimization of Curcumin from *Curcuma xanthorrhiza* Roxb. with supercritical carbon dioxide using ethanol as a cosolvent. ACS omega. 2024;9(1):1251-1264; <https://doi.org/10.1021/acsomega.3c07497>.

Adams RP. Identification of essential oil components by gas chromatography/ quadrupole mass spectroscopy. J. Am. Soc. Mass Spectrom. 2005;16:1902–3; https:// doi. org/ 10. 1016/j. jasms. 2005. 07. 008.
